# Evaluation of heritability of β-eudesmol/hinesol content ratio in *Atractylodes lancea* De Candolle

**DOI:** 10.1186/s41065-020-00123-3

**Published:** 2020-03-11

**Authors:** Takahiro Tsusaka, Bunsho Makino, Ryo Ohsawa, Hiroshi Ezura

**Affiliations:** 1 Graduate School of Life and Environmental Sciences, University of Tsukuba, Tsukuba, Ibaraki Japan; 2 Botanical Raw Materials Production Department 2, Tsumura & Co., Ami Town, Ibaraki Japan; 3 Botanical Raw Materials Research Laboratories , Tsumura & Co., 3586 Yoshiwara, Ami-machi, Ibashiki-gun, Ami Town, Ibaraki Japan; 4 Faculty of Life and Environmental Sciences, University of Tsukuba, Ten-nodai 1-1-1, Tsukuba, Ibaraki 305-8572 Japan

**Keywords:** *Atractylodes lancea*, β-Eudesmol, Hinesol, Content ratio, Heritability, Genotype–environment interaction

## Abstract

**Background:**

*Atractylodes lancea* De Candolle is a medicinal plant distributed in East Asia. Its rhizome has been used as an important crude drug in traditional Chinese and Japanese medicines for the treatment of numerous diseases and disorders. In recent years, the demand for mass production of the crude drug with a stable quality has increased. Its major active compounds are sesquiterpenoids, such as β-eudesmol and hinesol that have closely related chemical structures with each other. As the criteria for evaluating the quality of *A. lancea*, the β-eudesmol/hinesol content ratio is considered important. In *A. lancea*, the ratio could be considered to be influenced by genetic factors, geographical environment factors and these interactions. Few studies of a detail genetic analyses for β-eudesmol/hinesol content ratio have been reported. Therefore, we evaluated the heritability and genotype–environment interaction on the β-eudesmol/hinesol content ratio in *A. lancea* using clonal lines propagated with division of rhizome.

**Results:**

The heritability of the β-eudesmol/hinesol content ratio in *A. lancea* was evaluated through the cultivation of clonal lines of *A. lancea* in both different years (2016, 2017) and locations (Hokkaido, Ibaraki). Correlations between β-eudesmol and hinesol contents were identified in all clonal lines, with high correlation coefficients (r = 0.73–0.99). The broad-sense heritability of the β-eudesmol/hinesol content ratio was revealed to be high at 0.92. The effects of cultivation year were smaller than that of genotype, and few genotype–environment interactions were observed. In addition, the influence of cultivation location was also smaller than that of genotype, and the correlation between the two cultivation locations on the β-eudesmol/hinesol content ratio was high. The results suggested that the β-eudesmol/hinesol content ratio in *A. lancea* is highly dependent on genetic factors.

**Conclusion:**

We demonstrate that the heritability of β-eudesmol/hinesol content ratio is high and that the effects of genetic factors were stronger than that of environmental factors such as cultivation location and year. Our findings suggested that selective breeding and clonal propagation are effective strategies for the production of *A. lancea* with stable qualities for use in the production of crude drugs.

## Background

*Atractylodes lancea* De Candolle belonging the *Compositae* family is a perennial medicinal plant widely distributed across East Asia. Its dried rhizome has been used as an important crude drug in traditional Chinese and Japanese medicines [1]. In these medicines, various decoctions containing the crude drug have been used for a treatment of digestive disorders and body fluid imbalance [2, 3]. In modern pharmacological studies, the major active compounds obtained from *A. lancea* rhizomes have been shown to exhibit pharmacological activities on nervous, cardiovascular, and gastrointestinal systems [4]. Additionally, their anticancer, anti-inflammatory, and antimicrobial activities have also been reported [4]. Major active compounds in *A. lancea* rhizome are sesquiterpenoids such as β-eudesmol and hinesol, which have closely related chemical structures from each other [5]. Pharmacological activities of these two compounds are resembled, however intensity of pharmacological activity and action mechanism may be different [6]. For instance, both of β-eudesmol and hinesol have mitigation effects against gastric ulcer, but their pharmacological action mechanisms are different [6–8]. Additionally, hinesol has activity to induce apoptosis in human leukemia HL-60 cells, suggesting the possibility that hinesol may be useful anticancer drug, while, it is weak pharmacological activity in β-eudesmol [9]. In recent years, the demand for mass production of the crude drug with a stable quality has increased [10]. Therefore, keeping the contents of β-eudesmol and hinesol constant in *A. lancea* rhizomes is important to stabilize its pharmacological activity [11].

In the classical medicine texts, especially in Japan, it has also been mentioned that the crude drug highly suited for medicinal use deposits white cotton-like crystals on a section or epidermis of the dried rhizome [12]. Most of the crystal is comprised of β-eudesmol and hinesol as major constituents [5]. In addition, the formation of the crystal has been shown to be dependent on not only their high absolute contents but also an equivalent ratio of their contents in the dried rhizome [13]. Consequently, the β-eudesmol/hinesol content ratio is also assumed as an important quality criteria of *A. lancea* rhizomes as the crude drug suited for medicinal use [13, 14].

The quality of *A. lancea* rhizome is closely related to environment of its natural habitats, and β-eudesmol and hinesol content vary across geographical regions [13, 15]. We previously demonstrated that β-eudesmol and hinesol content are strongly influenced by genetic factors [16]. In addition, Takeda et al. [14] suggested that geographical differences in terms of the β-eudesmol/hinesol content ratio are mainly caused by genetic differences. Hence, detailed genetic analyses of broad-sense heritability, genotype–environment (G × E) interactions, and the effects of environmental factors on the β-eudesmol/hinesol content ratio are warranted.

In this study, we analyzed data from a previous study [16] on the β-eudesmol/hinesol content ratio in *A. lancea* rhizomes and investigated the heritability of this ratio. In particular, a total of 25 clonal lines of *A. lancea* were grown in an experimental field, and broad-sense heritability of the β-eudesmol/hinesol content ratio was estimated. Additionally, to investigate stabilities of the traits in annual variability and cultivation locations, we evaluated G × E interactions between genotype and cultivation year or location. Six clonal lines were grown under different years and locations, and two-way ANOVA and correlation analysis between different cultivation years or locations on the β-eudesmol/hinesol content ratio were performed. In the present study, we attempted to determine the relative effects of genetic factors and environmental factors on the β-eudesmol/hinesol content ratio in *A. lancea*.

## Results

### Estimation of broad sense heritability on the β-eudesmol/hinesol content ratio

**Fig. 1:**
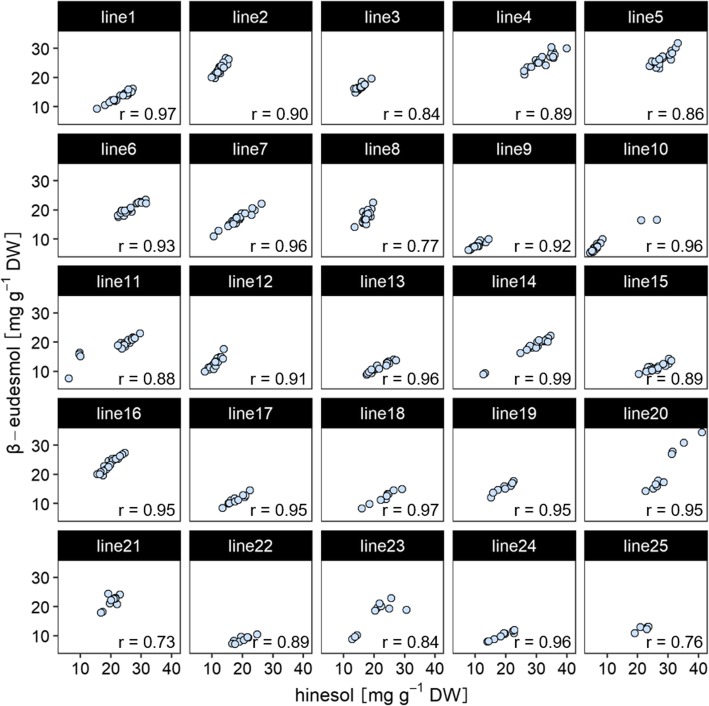
Correlation between β-eudesmol and hinesol contents in *A. lancea* clonal lines. Pearson’s correlation coefficients (r) were calculated for each clonal line. The number of replicates for each clone is as follows; lines 1–17 (*n* = 20), lines 18–24 (*n* = 10), and line 25 (*n* = 5)

In order to evaluate the heritability of the β-eudesmol/hinesol content ratio in *A. lancea*, 25 clonal lines were cultivated in an experimental field located in Ibaraki Prefecture (Japan), and the contents of the compounds were determined. Figure 1 shows that the results of correlation analysis between β-eudesmol and hinesol content in each clonal line. The range of β-eudesmol contents in all clonal lines were 5.3–34.5 mg / g, and the range of hinesol contents were 4.4–41.1 mg / g. As seen, β-eudesmol and hinesol content are significantly and positively correlated, with high r-values of 0.73–0.99 in all clonal lines, suggesting β-eudesmol/hinesol content ratio is stable within a clonal line.

**Fig. 2:**
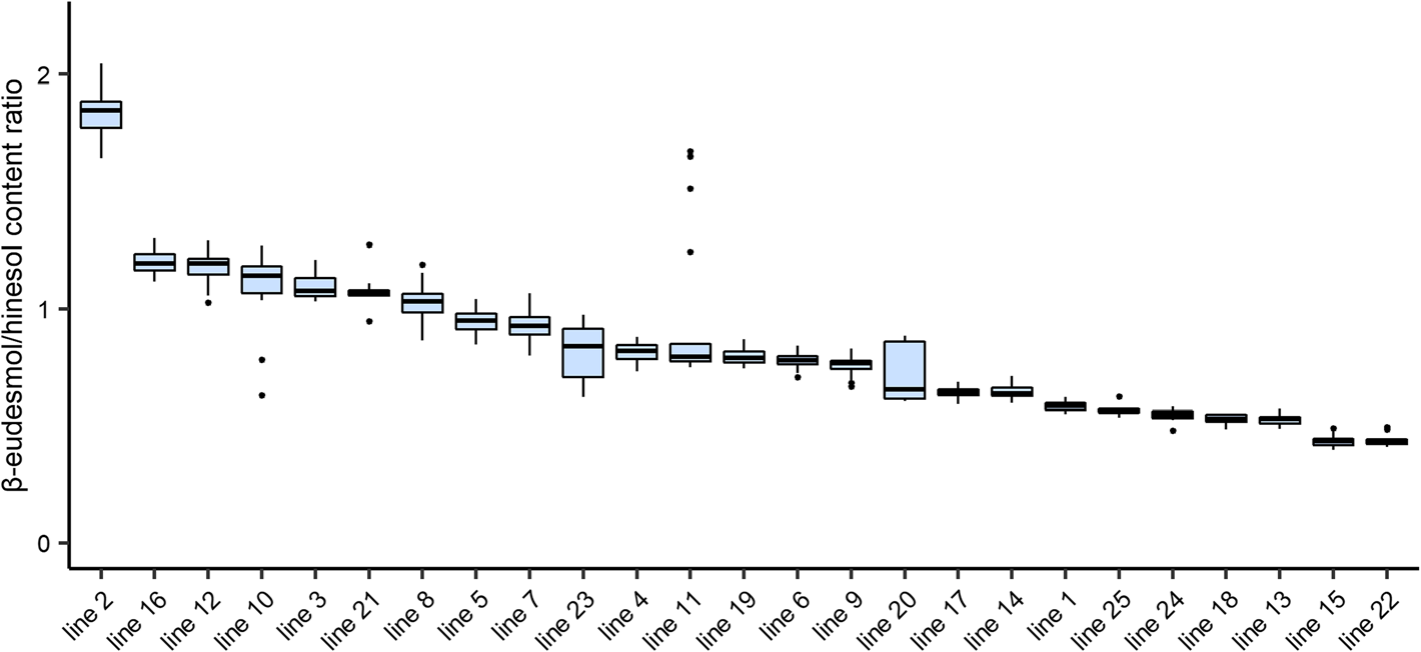
Range of variations in the β-eudesmol/hinesol content ratio in *A. lancea*. Boxes represent 25th–75th percentiles and middle lines represent medians. The vertical lines extend from minimum to maximum values. The numbers of biological replicates for each clonal line is as follows: lines 1–17 (n = 20), lines 18–24 (n = 10), and line 25 (n = 5)

Figure 2 shows variations in the β-eudesmol/hinesol content ratio across different *A. lancea* clonal lines. The ranges of variation for the β-eudesmol/hinesol content ratio within an *A. lacnea* clonal line were smaller than varietal differences (Fig. 2). One-way analysis of variance (ANOVA) identified significant differences (*P* > 0.01) in the β-eudesmol/hinesol content ratio among the *A. lancea* clonal lines (Table 1). From the ANOVA result, the broad-sense heritability of the β-eudesmol/hinesol content ratio was also revealed to be high at 0.92 (Table 1).

**Table 1:** Broad-sense heritability of the β-eudesmol/hinsol content ratio in *A. lancea*

	Df	Mean Sq	*P*-value	Effective replication	Genotypic variance	Environmental variance	Broad-sense heritability
Clonal line	24	1.71	<2e-16^a^	16.3	0.10	0.009	0.92
Residuals	390	0.01					

*Df* degree of freedom, *Mean Sq* Mean square; ^a^*P* < 0.01

### Effects of cultivation year on the β-eudesmol/hinesol content ratio

**Fig. 3:**
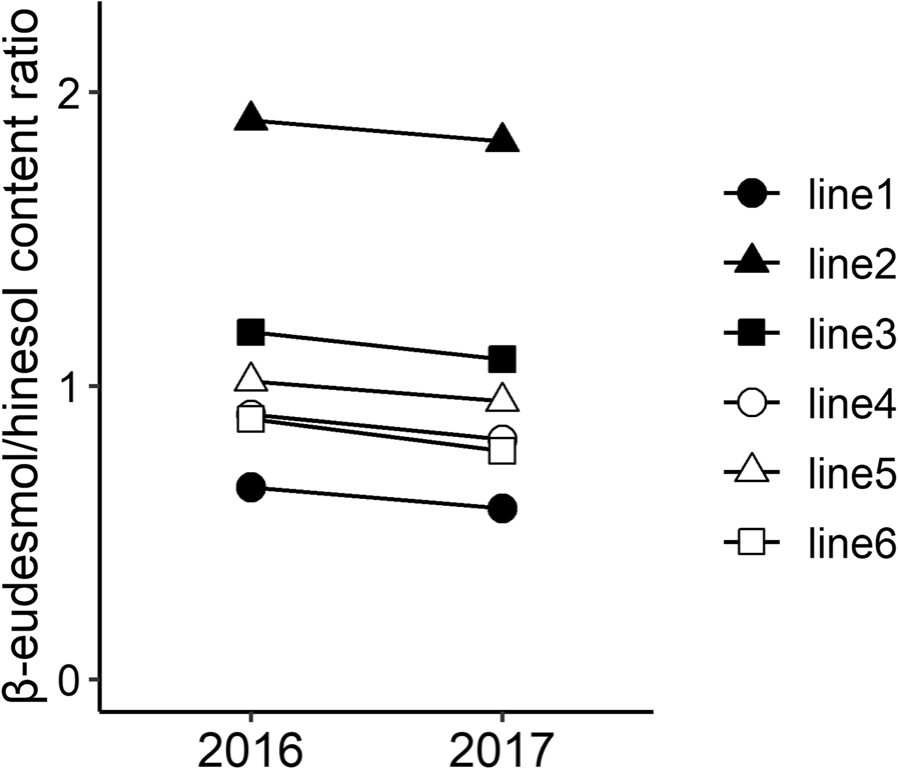
Interaction plots for interannual variability in the β-eudesmol/hinesol content ratio in *A. lancea*. Each point represents the mean of 20 measurements for all *A, lancea* clonal lines in 2016 and 2017

**Fig. 4:**
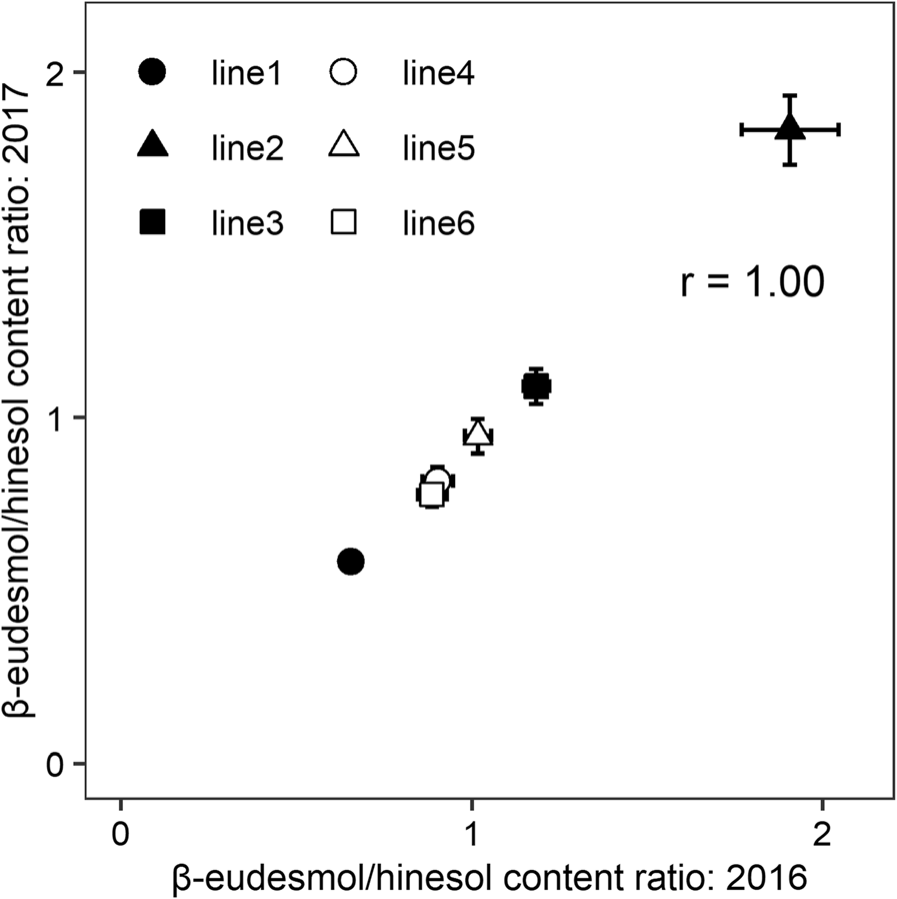
Correlation of the β-eudesmol/hinesol content ratio in *A. lancea* clonal lines grown in 2016 and 2017. Each point represents the mean of 20 measurements in 2016 and 2017, whereas bars indicate standard deviations

To determine the effects of cultivation year on the β-eudesmol/hinesol content ratio in *A. lancea* rhizome, 6 clonal lines were grown and analyzed in 2016 and 2017 under the same experimental field located in Ibaraki Prefecture. In two-way ANOVA of the β-eudesmol/hinesol content ratio, the effects of genotype (G) and cultivation year (Y) were significant, while mean square value for cultivation year was lower than that for genotype (Table 2). No significant differences in G × Y interactions were identified (Table 2). In addition, the broad-sense heritability of the β-eudesmol/hinesol content ratio, which was calculated using variance components from two-way ANOVA, was high at 1.00 (Table 2). Few qualitative interactions were observed between genotype and cultivation year (Fig. 3), and the correlation between the two cultivation years on the β-eudesmol/hinesol content ratio was high (r = 1.00; Fig. 4).

**Table 2:** Two-way ANOVA of β-eudesmol/hinsol content ratio in *A. lancea* lines grown in 2016 and 2017

	Df	Mean Sq	P-value	Variance components	Broad-sense heritability
Year (Y)	1	0.41	<2e-16^a^		1.00
Genotype (G)	5	7.63	<2e-16^a^	0.19	
G × Y interaction	5	0.002	0.70	−0.0001	
Residuals	228	0.004		0.004	

*Df* degree of freedom, *Mean Sq* Mean square; ^a^*P* < 0.01

### Effects of cultivation location the β-eudesmol/hinesol content ratio

In order to evaluate the effect of cultivation location on the β-eudesmol/hinesol content ratio, 6 clonal lines were grown at two different locations, Hokkaido and Ibaraki prefecture. Environmental condition of these cultivation locations were differed. Hokkaido is located in northernmost point in Japan, whereas Ibaraki prefecture is located in middle of Japan. The experimental field in Hokkaido was located in 43°.01′N, 140°.53′E, 8 m altitude, whereas the experimental field in Ibaraki prefecture was located in 35°.99′N, 140°.20′E, 25 m altitude. Mean temperatures for the cultivation period in Hokkaido and Ibaraki prefecture were 15.4 °C and 8.3 °C, respectively. Additionally, soil type and soil texture of 2 cultivation locations were differed. The soil type and soil texture in Ibaraki prefecture were andosol and loam, and that in Hokkaido were alluvial soil and sandy loam.

**Fig. 5:**
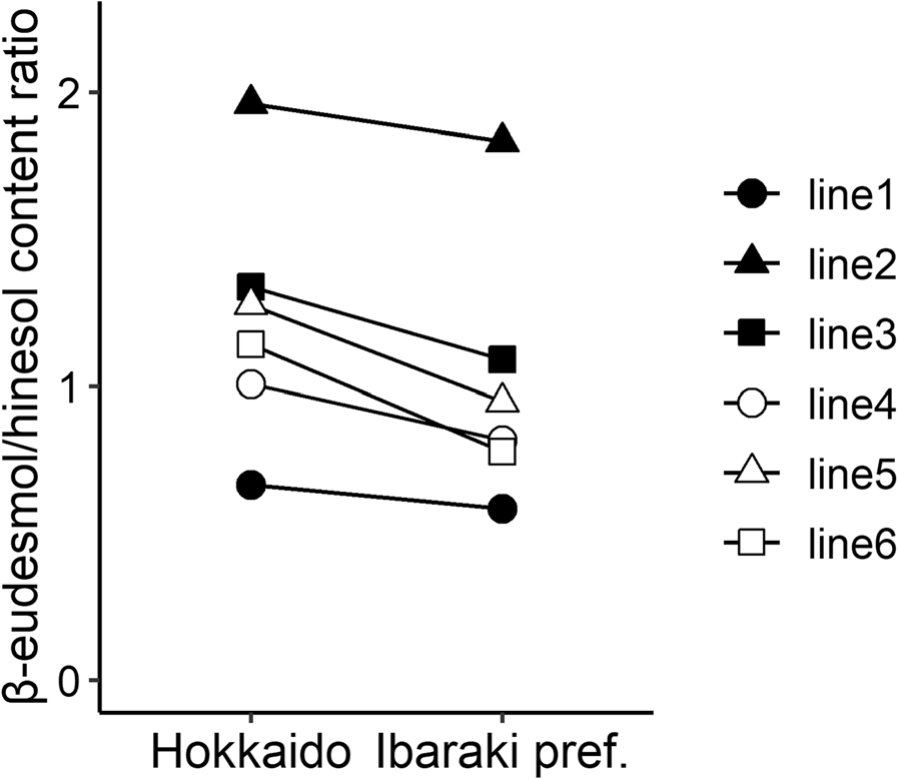
Interaction plots of β-eudesmol/hinesol content ratio in *A. lancea* lines grown in 2 cultivation locations. Data are presented as the mean of biological replicates for *A. lancea* clonal lines grown in Hokkaido; lines 1, 4, and 6 (n = 20), line 2 (*n* = 12), line 3 (*n* = 8), and line 5 (*n* = 18). Twenty replicates were generated for each *A. lancea* clonal lines grown in Ibaraki Prefecture

**Fig. 6:**
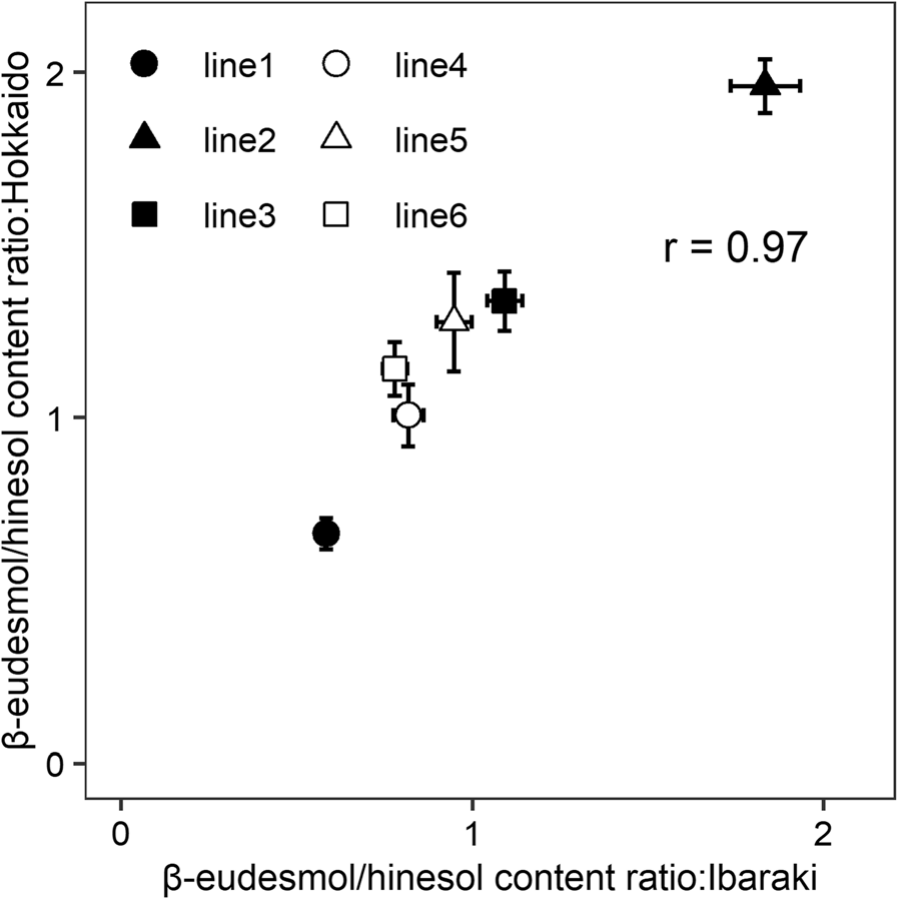
Correlation of β-eudesmol/hinesol content ratio in *A. lancea* lines grown in 2 cultivation locations. Data are presented as mean and standard deviation. The number of biological replicates for all *A. lancea* clonal lines grown in Hokkaido is as follows: lines 1, 4, and 6 (n = 20); line 2 (n = 12); line 3 (n = 8); and line 5 (n = 18). Twenty replicates were generated for each *A. lancea* clonal line grown in Ibaraki Prefecture

Two-way ANOVA of the β-eudesmol/hinesol content ratio identified significant differences for the cultivation location in terms of genotypes (G), cultivation locations (L), and G × L interaction; however, mean square for cultivation location and G × L interaction were smaller than that for genotype (Table 3). Additionally, broad-sense heritability of the β-eudesmol/hinesol content ratio was high at 0.98 in two-way ANOVA of variance components (Table 3). Furthermore, a minimal qualitative interaction was observed between genotype and cultivation location (Fig. 5), and the correlation coefficient for the β-eudesmol/hinesol content ratio between the two cultivation locations was 0.97 (Fig. 6).

**Table 3:** Two-way ANOVA of β-eudesmol/hinsol content ratio in *A. lancea* lines grown in 2 cultivation locations

	Df	Mean Sq	*P*-value	Variance components	Broad-sense heritability
Location (L)	1	1.21	<2e-16^a^		0.98
Genotype (G)	5	6.44	<2e-16^a^	0.17	
G × L interaction	5	0.12	<2e-16^a^	0.01	
Residuals	206	0.01		0.01	

*Df* degree of freedom, *Mean Sq* Mean square; ^a^*P* < 0.01

## Discussion

In this study, β-eudesmol content was positively correlated with hinesol content in all *A. lancea* clonal lines (Fig. 1). Takeda et al. [14] similarly showed a strong correlation between β-eudesmol and hinesol content in *A. lancea* clonal lines. Additionally, variations of the β-eudesmol/hinesol content ratio within an *A. lancea* clonal line were smaller than those among varietals (Fig. 2), and broad-sense heritability of the β-eudesmol/hinesol content ratio was high (Table 1), suggesting strong effects of genetic factors. These data indicate that selective breeding is an effective strategy for stabilizing the β-eudesmol/hinesol content ratio.

In analyses of the β-eudesmol/hinesol content ratio, we found no interactions between genotype and cultivation year, and mean square for genotype was higher than that for cultivation year (Table 2). In addition, we demonstrated high broad-sense heritability of the content ratio (Table 2), and a high positive correlation between the two cultivation years (Fig. 4). These results indicate that proportion of genetic variation in total variance is higher than annual variation, and the β-eudesmol/hinesol content ratio remains stable irrespective of interannual differences in environmental conditions.

The two-way ANOVA result for the comparative study of cultivation location identified significant differences in not only genotypes but also cultivation locations and G × L interaction (Table 3). Also, variations of cultivation location and G × L interaction were higher than that of cultivation years and G × Y interaction (Table 3). These results might be caused by variation of environmental conditions such as average temperature and soil conditions [16]. The environmental conditions of two cultivation locations were more varied than that of two cultivation years [16]. However, the broad-sense heritability and correlation coefficient between the two cultivation locations was exhibited high-values (Table 3 and Fig. 6), suggesting that the ratio of genetic variation in total variance is relatively larger than that of the environmental variation (cultivation location). These data indicate that the β-eudesmol/hinesol content ratio has wide adaptability in *A. lancea*.

In our previous study of the same samples, we determined the effects of environmental factors on β-eudesmol and hinesol content [16]. We showed that the broad-sense heritability of this ratio was higher than that of absolute β-eudesmol and hinesol content, and that the effects of cultivation year and location on the β-eudesmol/hinesol content ratio were smaller than on β-eudesmol and hinesol content [16].

In general, sesquiterpenoids are induced by plant hormones produced in response to biotic and abiotic stresses [17]. In *A. lancea*, β-eudesmol and hinesol production is reportedly induced by plant hormones, such as jasmonic acid and absisic acid, through symbiosis with endophytes [18, 19]. Soil acidity was shown to induce β-eudesmol accumulation in a previous study on *A. lancea* rhizomes [20]. These investigations show that absolute β-eudesmol and hinesol content vary with environmental factors. In contrast, we showed limited effects of environmental factors on the β-eudesmol/hinesol content ratio, corresponding to high broad-sense heritability of this ratio. β-Eudesmol and hinesol have the same chemical structure and molecular weight; therefore they likely have closely related biosynthetic pathways [5]. Hence, it is possible that the β-eudesmol/hinesol content ratio is genetically controlled in *A. lancea*. Recent transcriptome analyses of *A. lancea* identified several candidate genes related to sesquiterpenoid biosynthesis [11, 21]. Additionally, high levels of single- nucleotide polymorphisms have been detected in cDNA libraries of *A. lancea* leaf, stem, and root tissues [22]. These findings highlight the possibility of developing a marker-assisted selection strategy based on the β-eudesmol/hinesol content ratio using genetic association analysis.

Herein, we indicate that the β-eudesmol/hinesol content ratio in *A. lancea* rhizomes is highly dependent on genetic factors and suggest that clonal propagation is effective for stabilizing this ratio in *A. lancea*. *A. lancea* tissues have been propagated in vitro, particularly tip tissues have been cultured extensively [23]. In particular, Shoyama et al. [24] indicated that 6-benzylaminopurine facilitates shoot propagation, and gibberellin stimulates root enlargement in *A. lancea*. Thus, *A. lancea* strains with a stable β-eudesmol/hinesol content ratio could be propagated through mass propagation.

## Conclusion

We demonstrate that the heritability of β-eudesmol/hinesol content ratio is high and that the effects of genetic factors were stronger than that of environmental factors such as cultivation location and year. Our findings suggest that selective breeding and clonal propagation are effective strategies for the production of *A. lancea* with stable qualities for use in the production of crude drugs. Further molecular biological analyses such as isplation of genes related to biosynthesis of sesquiterpenoids such as β-eudesmol and hinesol are warranted.

## Methods

### Plant materials

Twenty-five *A. lancea* clones (lines 1–25) were propagated from rhizomes of a single plant as described previously [16]. In brief, original seed of *A. lancea* were obtained from another previous study [25]. These seeds were originated from China. According to morphological characteristics, the plants were identified as *A. lancea* by the first author. Voucher specimens (THS33885) were deposited at the herbarium stock room of the Botanical Raw Materials Research Laboratories, Tsumura & Co. Japan.

### Cultivation of *A. lancea*

Plants were cultivated following the method described in a previous study [16]. To estimate the broad-sense heritability of the β-eudesmol/hinesol content ratio in *A. lancea* rhizomes, 25 clonal lines were grown in an experimental field in Ami Town, Inashiki-gun, Ibaraki Prefecture (35°.99′N, 140°.20′E), Japan, in 2017. Rhizomes of these *A. lancea* clonal lines were cut into 50-g sections; planted on November 25, 2016; and harvested on November 23, 2017. The number of biological replicate plants for each clonal line was as follows: lines 1–17 (*n* = 20), lines 18–24 (*n* = 10), line 25 (*n* = 5).

To evaluate G × E interaction and the effects of environmental factors, 6 clonal lines were grown in different years and in different locations. To examine the effects of cultivation year, 6 clonal lines (lines 1–6) were cultivated in 2016 in an experimental field located in Ibaraki Prefecture. We planted the six clones on November 25, 2015 and harvested them on November 23, 2016. These experiments were performed with 20 biological replicates for each clonal line. In addition, to evaluate the effects of location, six clonal lines were grown in 2017 in another experimental field at Kyowa-Town, Hokkaido (43°.01′N, 140°.53′E), Japan. Rhizomes of the 6 clonal lines were divided into 50 g sections and we cultivated the 6 clonal lines from October 21, 2016, to October 19, 2017, in Hokkaido. This experiment was performed with 8–20 biological replicates for each clonal line as follows: line 1 (n = 20), line 2 (*n* = 12), line 3 (*n* = 8), line 4 (n = 20), line 5 (*n* = 18), and line 6 (n = 20).

### Extraction of sesquiterpenoids and gas chromatography-mass spectrometry (GC-MS) analysis

β-Eudesmol and hinesol content in *A. lancea* rhizomes were determined as described previously [16]. The *A. lancea* rhizomes were dried in a convection drying oven (RY-120HG, ALP Co., Ltd., Japan) at 50 °C for 7 days and pulverized using a vibrating rod mill (TI-200, Cosmic Mechanical Technology, Co. Ltd., Japan) for GC-MS analysis. The powder samples of *A. lancea* rhizomes were accurately weighed at 0.5 g, and extracted with n-hexane (25 ml) using a recipro shacker (SR-1, TAITEC Co., Japan) for 15 min, followed by centrifugation (1660×*g*, 10 min). After collection of the supernatant, the residues were re-extracted with n-hexane (20 ml) in a same manner. An internal standard (I.S.), phenanthrene (1.5 mg, 1 ml in n-hexane), was added to the combined supernatants in 50 ml-volumetric flask, and the solutions were made up by adding n-hexane to a total volume of 50 ml. The analyses were conducted using an Agilent 7890A gas chromatograph (GC) coupled to a 5975C mass spectrometer (MS) (Agilent Technologies, Palo Alto, CA, USA). Sample solutions (1 μL) were injected into a DB-WAX capillary column (polyethylene glycol, 30 m × 250 μm *i.d.*, 0.25 μm film thickness; Agilent J&W Scientific, Folsom, CA) in a split ratio of 50:1. Helium was used as the carrier gas at a flow rate of 1 mL /min. The injector temperature was set at 240 °C. The column oven temperature was initially held at 160 °C for 2 min after injection, and programmed to increase from 160 to 200 °C at a rate of 5 °C /min, then increase from 200 to 240 °C at a rate of 8 °C /min, and hold at 240 °C for 5 min. The interface temperature was set at 240 °C. The MS was operated in an electron impact ionization at 70 eV and the temperatures of the ion source and the quadrupole mass spectrometer were set at 230 °C and 150 °C, respectively. Total ion current (TIC) chromatograms were acquired in a mass range of 40–500 amu using an Agilent MSD Chemstation software (version E.02.00.493).

The quantitative analyses of β-eudesmol and hinesol were calculated on the basis of peak-area ratio to the I.S. in TIC chromatogram and regression analyses were performed. Standards of β-eudesmol (14.08 mg) and hinesol (20.16 mg), provided by Tsumura & Co. (Japan), were initially dissolved in each 10 mL hexane, and then stepwise-diluted with hexane followed by adding the I.S. (1.5 mg, in 1 mL hexane) to make series of the standard solutions ranging in concentration from 0.005 to 0.7 mg /mL of each compounds. The correlation coefficients for the standards of β-eudesmol and hinesol were 0.99 and 1.00, respectively. The contents of the sesquiterpenoids were expressed based on the dry weight of the powdered sample.

### Statistical analysis

Statistical analyses were performed using R (version 3.5.0). To evaluate the effects of genetic factors on the β-eudesmol/hinesol content ratio, one-way ANOVA was performed using the data of *A. laneca* clonal line grown in an experimental field. Broad-sense heritability (h_B_) was calculated from variance components in ANOVA as follows: h_B_ = σ^2^_G_ / (σ^2^_G_ + σ^2^_E_), where σ^2^_G_ is genotypic variance and σ^2^_E_ is environmental variance [26, 27]. Effective numbers of replicates (r) for estimating σ^2^_G_ were calculated as follows: $r=\left(\sum \limits_{i=1}^a{r}_i-\sum \limits_{i=1}^a{r}_i^2/\sum \limits_{i=1}^a{r}_i\right)/\left(\mathrm{a}-1\right)$, where a is the number of clonal lines [28].

To assess G × E interaction and the effects of environmental factors, we performed analyses of the β-eudesmol/hinesol content ratio with two-way ANOVA and Pearson’s correlation tests as well as compared *A. lancea* grown in different years and at different locations. Further, broad-sense heritability was estimated from two-way ANOVA values using the following formula: h_B_ = σ^2^_G_ / [σ^2^_G_ + (σ^2^_G × E_ / e) + (σ^2^_E_ / re)], where σ^2^_G × E_ is the G × E interaction variance and e is the number of experiments (cultivation year or location) [29].

## Data Availability

The datasets supporting the conclusions and methods description are included within the article.

## Abbreviations

ANOVA: Analysis of variance
E: Environment
G × E: Genotype–environment interaction
G: Genotype
GC/MS: Gas chromatography/mass spectrometry
h_B_: Broad-sense heritability
L: Cultivation location
Y: Cultivation year
